# Identification of a Mechanical Rheostat in the Hydrophobic Core of Protein L

**DOI:** 10.1016/j.jmb.2009.08.015

**Published:** 2009-10-16

**Authors:** David P. Sadler, Eva Petrik, Yukinori Taniguchi, James R. Pullen, Masaru Kawakami, Sheena E. Radford, David J. Brockwell

**Affiliations:** 1 Astbury Centre for Structural Molecular Biology and the Institute of Molecular and Cellular Biology, University of Leeds, Leeds LS2 9JT, UK; 2 School of Materials Science, Japan Advanced Institute of Science and Technology, 1-1 Asahidai, Nomi, Ishikawa, Japan

**Keywords:** Ig, immunoglobulin, WT, wild type, GdmHCl, guanidinium hydrochloride, force microscopy, mechanical properties, protein engineering, unfolding, protein L

## Abstract

The ability of proteins and their complexes to withstand or respond to mechanical stimuli is vital for cells to maintain their structural organisation, to relay external signals and to facilitate unfolding and remodelling. Force spectroscopy using the atomic force microscope allows the behaviour of single protein molecules under an applied extension to be investigated and their mechanical strength to be quantified. protein L, a simple model protein, displays moderate mechanical strength and is thought to unfold by the shearing of two mechanical sub-domains. Here, we investigate the importance of side-chain packing for the mechanical strength of protein L by measuring the mechanical strength of a series of protein L variants containing single conservative hydrophobic volume deletion mutants. Of the five thermodynamically destabilised variants characterised, only one residue (I60V) close to the interface between two mechanical sub-domains was found to differ in mechanical properties to wild type (Δ*F*_I60V–WT_ = − 36 pN at 447 nm s^− 1^, Δ*x*_uI60V–WT_ = 0.2 nm). Φ-value analysis of the unfolding data revealed a highly native transition state. To test whether the number of hydrophobic contacts across the mechanical interface does affect the mechanical strength of protein L, we measured the mechanical properties of two further variants. protein L L10F, which increases core packing but does not enhance interfacial contacts, increased mechanical strength by 13 ± 11 pN at 447 nm s^− 1^. By contrast, protein L I60F, which increases both core and cross-interface contacts, increased mechanical strength by 72 ± 13 pN at 447 nm s^− 1^. These data suggest a method by which nature can evolve a varied mechanical response from a limited number of topologies and demonstrate a generic but facile method by which the mechanical strength of proteins can be rationally modified.

## Introduction

It is now clear that the application of mechanical force to biomolecules and their complexes plays a role in many diverse processes *in vivo*.^1,2^ To investigate how nature utilises force to catalyse these processes and to apply this knowledge to design novel proteins with tailored mechanical properties, it is necessary to understand the determinants of protein mechanical strength. To date, data from single-molecule mechanical unfolding experiments suggest that the mechanical strength of proteins can be ranked based on the type and topology of secondary structural elements relative to the positions at which force is applied.^3–5^ However, topological arguments cannot explain the large differences in mechanical strength observed for proteins within families such as the immunoglobulin (Ig)-like domains of titin^6^ or between protein L and protein GB1.^7,8^ For example, the topologies of protein L and protein GB1 are identical, yet protein L unfolds at a force 30 pN lower than that of protein GB1. As these proteins have low sequence identity (16%),^9^ this suggests that the mechanical properties of a protein are determined by the topology of the main chain but may be tailored by the precise packing of amino acid side chains within the hydrophobic core. The role that core residues play in determining mechanical strength is unclear. Most observations to date suggest that such residues play only a minor role,^10,11^ whilst some indicate that subtle rearrangement of the core can affect the observed unfolding force.^12,13^

**Fig. 1:**
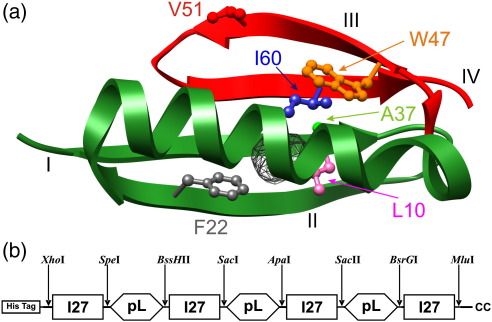
(a) Three-dimensional structure of protein L (PDB ID: 1HZ6^15^). Side chains of residues mutated in this study are shown as ball-and-stick representation (L10, pink; F22, grey; A37, green; W47, orange; V51, red; I60, blue). The main chain of residues that form mechanical sub-domain 1 (residues 1–44, comprising strand I, the β-hairpin, strand II and the helix) and sub-domain 2 (residues 45–64 comprising strand III, the second β-hairpin and strand IV) is coloured green and red, respectively. The cavity in the hydrophobic core of WT protein L is shown as a meshed surface. The cavity was identified using CASTp software,^16^ and the figure was drawn using Chimera.^17^ (b) Heteropolymeric construct used in this study. Unique pairs of restriction endonuclease sites (arrows) define each cassette of protein L (hexagons) or I27 (C47S, C63S) (rectangles). The hexa-histidine tag at the N-terminus allows facile purification, and two C-terminal cysteine residues are used to immobilise the protein onto a gold substrate.

We have previously used the topologically simple protein L to investigate the importance of topology in defining the mechanical strength of proteins. protein L is the B1 domain of the large multi-domain virulence factor Protein L, which is expressed on the surface of pathogenic bacteria. Protein L comprises tandem repeats of highly homologous domains that allow evasion of the host's immune system by multisite binding to a wide range of mammalian Igs in a nonantigenic manner.^14^ Using atomic force microscopy and simulation techniques, we showed that protein L unfolds in a single step by the shearing of two mechanical sub-domains (Fig. 1).^7^ More recently, Liu *et al.* used magnetic tweezers coupled with evanescent nanometry to investigate the behaviour of protein L under a low-force regime.^18^ Measurement of the unfolding kinetics under application of 13–120 pN yielded a similar intrinsic unfolding rate constant to that previously measured at the high-force regime, suggesting that a single barrier acts as the rate-limiting step to unfolding across a broad range of the dynamic force spectrum of this simple protein. Here, we investigate the structure of the rate-limiting transition state by quantifying the effect of a series of conservative hydrophobic deletion mutations on the unfolding force of protein L over a range of pulling speeds.

## Results

### Design and over-expression of concatenated protein L variants

A protein engineering approach was taken to assess the importance of specific side-chain interactions in distinct regions of protein L in determining its mechanical unfolding properties. The wild-type (WT) protein in this study is a pseudo-WT (Y47W) constructed by the Baker group to facilitate protein folding studies.^19^ Guided by thermodynamic and kinetic stability data published by the same group^20^ (see Table 1), we designed five variants, each containing a single conservative hydrophobic volume reduction mutation in one of the secondary structural elements of protein L (strand I: L10A, strand II: F22A, helix: A37G, strand III: V51A and strand IV: I60V, see Fig. 1a). Initially, pentameric homopolymers were constructed similar to those described previously for WT protein L.^7^ However, pentameric constructs of the most thermodynamically unstable variants (L10A, F22A and A37G) were found to express in an insoluble form, precluding their use for these studies. To obviate these problems, we created heptameric heteropolymers [((I27-pL^X^)_3_I27), where X denotes the variant] (Fig. 1b), comprised of three protein L domains interdigitated with four copies of the I27 mutant (C47S C63S).^21,22^ Heteropolymeric proteins provide several advantages for these studies in that the scaffold protein allows soluble over-expression of unstable or aggregation-prone proteins (probably due to the low sequence identity between each tandem pair^23^) and have a known mechanical response so that it can act both as a mechanical fingerprint and as an internal standard against which the effects of each protein L mutation can be benchmarked. Whilst construction of heteropolymers has proved successful for the soluble expression of other concatenated proteins,^24–26^ heptameric protein L variants were still found to be expressed in an insoluble form when induced using standard methods. Protein expression by auto-induction, however, yielded sufficient soluble protein for isolation using a standard two-step purification procedure (Materials and Methods).

### Investigating the mechanical effects of hydrophobic core reduction on protein L

**Fig. 2:**
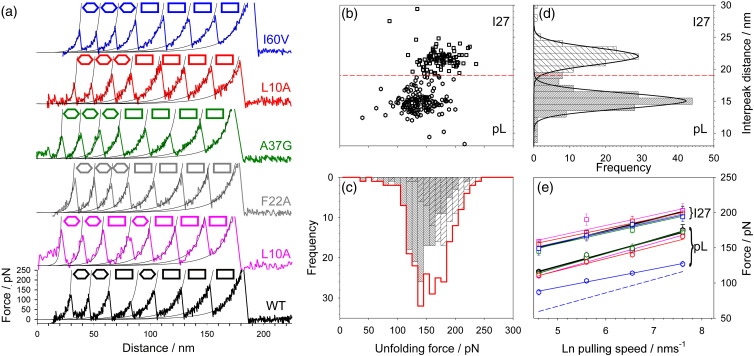
Summary of mechanical unfolding data for WT and variant protein L heteropolymers ((I27-pL^X^)_3_I27). (a) Sample force-extension data for WT (black), and the protein L variants L10A (pink), F22A (grey), A37G (green), V51A (red) and I60V (blue) within ((I27-pL^X^)_3_I27). Fitting the rising edge of successive unfolding events to a worm-like chain model for polymer elasticity (continuous lines) or measuring the distance between subsequent unfolding events allows identification of the type of domain being unfolded: protein L (hexagons) and I27 (rectangles). (b) Force–distance scattergram of one data set [((I27-pL^WT^)_3_I27) at a pulling speed of 700 nm s^− 1^, *n* = 236] reveals that, whilst distributions of unfolding force for protein L (hexagons) or I27 (squares) overlap significantly [see force–frequency histogram for all events, red line in (c)], little overlap is observed in the interpeak distance, allowing clear identification of protein L (grey) and I27 (open hatched) unfolding events (d). Events with interpeak distances of less than or greater than 19 nm, respectively, were binned into protein L and I27 data sets [red broken line in (b) and (d)]. (c) Force–frequency histogram for all events in this data set (red line) and when separated into protein L (fine hatches, mode 143 pN) and I27 (coarse hatches, mode 170 pN) unfolding events. (d) Distance–frequency histograms show a bimodal distribution centred in this case at 15.0 and 22.0 nm for protein L and I27, respectively. (e) Speed dependence of protein L variants and I27 domains within each heptamer (same colour scheme as above). Continuous lines are linear fits to the mean value of the most likely unfolding forces at 100, 270, 700 and 2000 nm s^− 1^ weighted by the reciprocal of the error for each point. Error bars shown for each data point are the standard error of multiple data sets. Blue broken line shows speed dependence of the unfolding force for a hypothetical protein L variant that unfolds over the same transition state as WT protein L with a Φ_F_^F^ = 0 but the same thermodynamic stability as protein L I60V.

Single-molecule mechanical unfolding using atomic force microscopy^27^ was used to characterise the effects of each mutation on the mechanical properties of protein L. Sample ‘sawtooth’ force-extension profiles of heptamers containing WT protein L ((I27-pL^WT^)_3_I27) and its variants ((I27-pL^X^)_3_I27) are shown in Fig. 2a, where each ‘tooth’ reports on the unfolding of a single domain of either protein L or I27. Each force-extension profile describes unfolding events for protein L and I27, and examination of a sample force–frequency histogram (Fig. 2c) reveals a single degenerate distribution. However, measurement of the interpeak distance (*L*_i_) or fitting worm-like chain models to sequential unfolding events yields two populations with *L*_i_ values of 15.7 ± 1.4 and 23.0 ± 1.6 nm (Fig. 2d) and an increase in contour length upon unfolding of 18.0 ± 1.3 and 25.6 ± 1.0 nm. These values reflect the difference in size of the force-resistant structures for protein L and I27 (60 and 81 residues, respectively) and are in accord with values previously published for protein L and I27.^7,28^ The spacing between each event thus allows each to be assigned to a protein domain type, allowing resolution of force–frequency histograms for each domain type within the heteropolymer (Fig. 2c). The force–distance scattergram (Fig. 2b) and interpeak distance–frequency histogram typically display two well-resolved populations with a minimum at ∼ 19 nm. In the example shown (Fig. 2b–d), overlap in the fitted distributions does not allow the identity of a small number of unfolding events to be assigned. Analysis of data sets obtained for ((I27-pL^WT^)_3_I27), ((I27-pL^I60V^)_3_I27) and ((I27-pL^I60F^)_3_I27) demonstrates that removal of these unassignable events (105 out of 5350 events) does not change the unfolding force significantly (0.23 ± 0.07% of modal force value). An interpeak distance of 19 nm can thus be used to bin the data, allowing the unfolding force for each domain type to be quantified. Performing this process at pulling speeds of 100, 270, 700 and 2000 nm s^− 1^, using previously published protocols^7,21,29^ (Materials and Methods), allows the speed dependence of the unfolding force to be assessed from which the basic features of the energy landscape of unfolding (*x*_u_, the distance to the unfolding transition state, and *k*_u_^0F^, related to the height of the barrier traversed during mechanical unfolding) can be determined and the effects of mutation on these parameters quantified.^5,30^ A summary of the unfolding data obtained for all variants can be found in Supplementary Information.

**Fig. 3:**
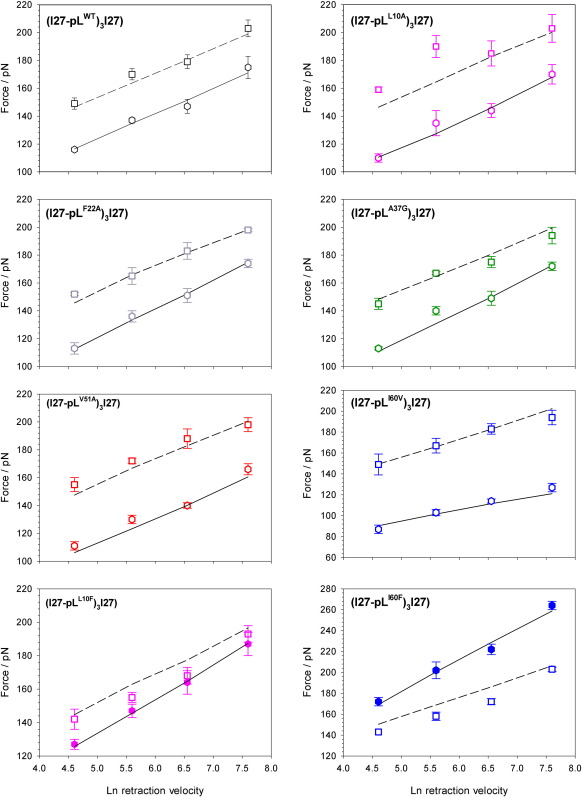
Monte Carlo fitting of the speed dependence of the unfolding force for ((I27-pL^WT^)_3_I27) and all protein L variants. For each ((I27-pL^X^)_3_I27) construct, average modal unfolding forces obtained by experiment are shown by hexagons for protein L and squares for I27. The simulated force dependence using fixed values of *k*_u_ and *x*_u_ for I27 and fitted values of *k*_u_ and *x*_u_ for protein L obtained as described (Materials and Methods) is shown as broken and continuous lines, respectively.

Of the five variants analysed, four unfolded at forces similar to that of WT protein L (Fig. 2e). By contrast, protein L I60V unfolded at a markedly lower force (Δ*F*_MUT–WT_ = − 36 pN at 447 nm s^− 1^) (Fig. 2e) than all the other variants studied, despite its modest thermodynamic destabilisation (Table 1). In addition, the speed dependence of the unfolding force of protein L I60V differs significantly from the other variants whilst that for I27 within ((I27-pL^I60V^)_3_I27) is identical with the other variants (Fig. 2e). Direct interpretation of these results is complicated by the effect of unfolding history^22^ as the most likely unfolding force for any domain within a heteropolymer depends on the instantaneous loading rate applied onto that domain before failure. As each unfolding event changes the forced loading rate (by changes in compliance), mutations that alter the unfolding sequence of each domain type within the heteropolymer may alter the apparent mechanical strength of each domain type. Consequently, Monte Carlo simulations (Materials and Methods) were used to obviate these effects and to estimate values for *k*_u_^0F^ and *x*_u_ (Table 1 and Fig. 3). These calculations revealed that *x*_u_ for I60V is significantly larger than that of WT protein L and the other variants (0.46 nm *versus* 0.22–0.27 nm, respectively), indicating that the transition state to unfolding for this variant occurs further from the native state. Whilst interpreting *x*_u_ in structural terms is complex,^31^ the larger *x*_u_ for protein L I60V suggests that this variant is ‘softer’ than other variants; that is, it undergoes greater deformation prior to global unfolding.

### Mechanical Φ−value analysis reveals a highly native-like transition state

By comparing the relative effects of mutation on the ground and transition states of mechanical unfolding, it is possible to estimate the extent of native structure preservation in the mechanical unfolding transition state by calculating ‘Φ values’.^32,33^ A folding Φ value is the ratio between the change in stability of the transition state (TS) to folding from the unfolded (U) to native (N) state (ΔΔ*G*°_U–TS_) and the change in thermodynamic stability of the native state (ΔΔ*G*°_UN_):

$$\Phi_{F}=\frac{\Delta \Delta G_{U-\text{TS}}^{{}^{\circ}}}{\Delta \Delta G_{\text{UN}}^{{}^{\circ}}}=1-\frac{\Delta \Delta G_{\text{TS}-N}^{{}^{\circ}}}{\Delta \Delta G_{\text{UN}}^{{}^{\circ}}}$$

In this method, ΔΔ*G*_UN_ is calculated by measuring the thermodynamic stability of each variant relative to the WT and, thus, assumes that the folded ground states for mechanical and chemical denaturation are of similar energy. Mechanical unfolding data obtained in this study and in previous studies^7,18^ show no evidence of any intermediates in the unfolding of WT protein L or its variants. The effect of mutation on the unfolding transition state (ΔΔ*G*_TS–N_) can either be calculated directly from changes in the mean unfolding force or, in cases where such data are complicated by loading rate effects, indirectly from *k*_u_^0F^ values extracted from MC fitting of the speed dependence of the unfolding force.^33^ Both types of analysis were performed (Materials and Methods), and the results are shown in Table 1. For ease of comparison, folding Φ values are discussed here, which describe the transition state for force folding (Φ_F_^F^) or the transition state for folding determined previously by chemical denaturants (Φ_F_^D^). Strikingly, Φ_F_^F^ for L10A, F22A, A37G and V51A was found to be close to 1 (Table 1). Although the difference of *x*_u_ observed for I60V precludes direct comparison with the other variants (see blue broken line, Fig. 2e), Φ_F_^F^ = 0.33 ± 0.08 for I60V, suggesting that the native structure is significantly perturbed at this location in the mechanical TS. These data describe a TS for unfolding that is highly native-like: application of force simply displaces strand IV, consistent with previous predications made by molecular dynamics simulations.^7^ It is interesting to note that whilst the mechanical destabilisation for V51A is just within the error of the WT data (Δ*F* = − 8 ± 10, Φ_F_^F^ = 0.78 ± 0.12), the data are consistently lower than WT values, suggesting that the displacement of strand IV may be propagated through the β-hairpin in the mechanical transition state. In contrast with the high Φ_F_^F^ values determined here, Φ_F_^D^ values (Table 1) reveal that native protein structure is highly perturbed at all of these sites in the folding TS probed by chemical denaturants. This observation underlines previous findings that mechanical and chemical denaturants induce unfolding *via* different pathways,^10,34^ emphasising the distinct methods by which force (local denaturant) and chaotropes (global denaturant) unfold proteins.

### Rational design of protein L variants with enhanced mechanical strength

**Fig. 4:**
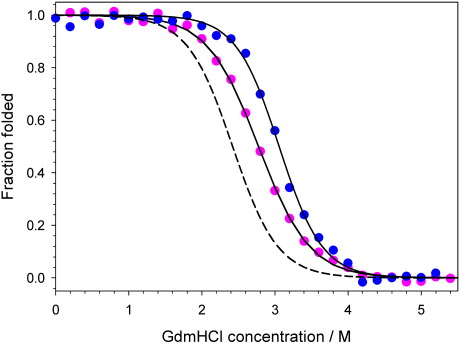
Equilibrium denaturation of monomeric protein L variants (L10F, pink circles; I60F, blue circles) followed by tryptophan fluorescence. Fitting each data set to a two-state model (continuous lines) yields an *m* value of 7.1 and 8.3 kJ mol^− 1^ M^− 1^ and a thermodynamic stability of 19.6 and 25.4 kJ mol^− 1^ for L10F and I60F protein L, respectively. For comparison, the equilibrium denaturation curve for the WT protein L (Y47W) used in this study is shown calculated using previously published parameters (broken line).^19^

**Fig. 5:**
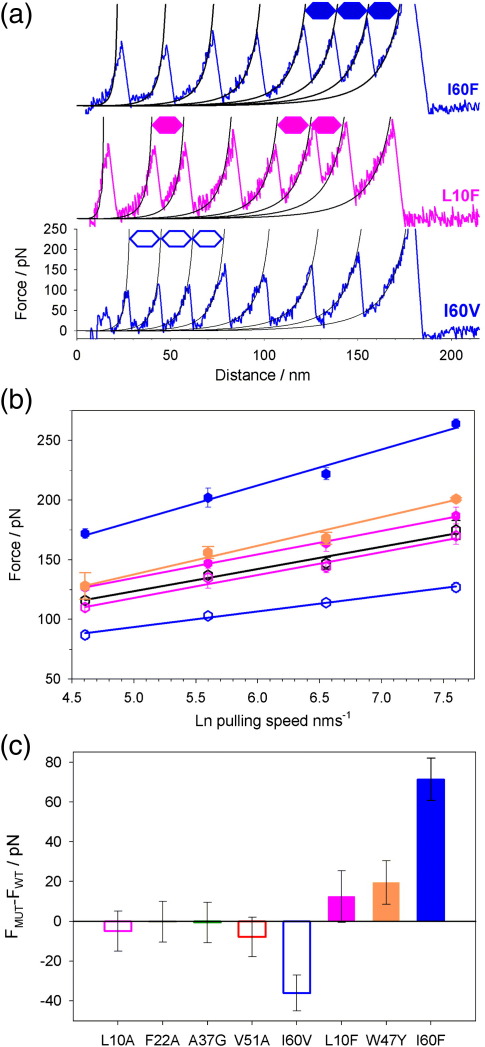
Mechanical unfolding data for rationally designed variants of protein L. (a) Sample force-extension profiles for ((I27-pL^I60V^)_3_I27), ((I27-pL^L10F^)_3_I27) and ((I27-pL^I60F^)_3_I27). protein L unfolding events are identified by hexagons. (b) Speed dependence of unfolding force for hydrophobic enhancement variants ((I27-pL^I60F^)_3_I27) (filled, blue) and ((I27-pL^L10F^)_3_I27) (filled, pink) with their analogous hydrophobic deletion variants ((I27-pL^I60V^)_3_I27) (open, blue) and ((I27-pL^L10A^)_3_I27) (open, pink). WT protein L ((I27-pL^WT^)_3_I27) (open, black) and the true ‘reverted’ WT variant protein L ((I27-pL^W47Y^)_3_I27) (filled, orange) are also shown. (c) Core mutations can be used to modulate the mechanical strength of protein L. Bar chart showing the change in mechanical strength of different pL variants at 447 nm s^− 1^ relative to the WT protein. Error bars were propagated from a percentage error of each unfolding force based on replicate measurements of I27 unfolding data.

The observation that removal of a single methylene group from I60 in the core of protein L results in a marked reduction in the unfolding force suggests that it may be possible to enhance the mechanical stability of protein L by increasing the number of force-bearing interactions at this site that make contacts with residues in the second sub-domain. Conversely, a similar increase in hydrophobic contacts made by increasing the hydrophobic volume of an amino acid side chain that does not make contacts with both sub-domains would be expected to have little effect on the mechanical stability of protein L. The effect of insertion of larger hydrophobic side chains into the core of a protein depends on whether the existing hydrophobic contacts of the WT at the mutation site are optimally (destabilising) or more loosely packed (stabilising).^35^ Fortuitously for this study, creation of the pseudo-WT (Y47W protein L) resulted in the formation of a small cavity (∼ 30 Å^3^) lined by several residues probed in this study (L10, F22, A37 and I60).^15^ Furthermore, phage display selection for thermal stability from a heavily mutated combinatorial library of protein L Y47W revealed a preference for larger nonpolar side chains at some of these cavity-surrounding sites (A8, L10, A33 and I60).^15,36^ These observations allowed the design of two variants with enhanced hydrophobic contacts that were expected to have distinctive mechanical phenotypes. protein L L10F was selected to fill the cavity but not enhance inter-sub-domain interactions, whilst protein L I60F was selected to both fill the cavity and enhance these interactions. The effect of these mutations on the thermodynamic stability of protein L was assessed by equilibrium denaturation of monomeric analogues of each variant (Materials and Methods). As expected, both variants showed increased thermodynamic stability relative to WT protein L (Fig. 4 and Table 1). After construction and purification of ((I27-pL^L10F^)_3_I27) and ((I27-pL^I60F^)_3_I27), the mechanical properties of each variant were characterised as described for the hydrophobic deletion variants above. Examination of sample force-extension profiles and the speed dependence of the unfolding force show that each mutation results in a distinct mechanical phenotype (Fig. 5a and b). Whilst protein L L10F shows only a modest increase in mechanical strength (Δ*F*_MUT–WT_ = 13 ± 11 pN at 447 nm s^− 1^), increasing the hydrophobic volume at position 60 increases the mechanical strength by a striking 72 ± 13 pN (Fig. 5c). A final variant that reverted to the true WT of protein L [W47Y protein L, ((I27-pL^W47Y^)_3_I27)] was characterised in order to obviate the possibility that this enhancement resulted simply from more efficient hydrophobic packing of the core. Similarly to L10F, this construct was found to display only a moderate increase in mechanical strength (Δ*F*_W47Y–WT_ = 20 ± 11 pN at 447 nm s^− 1^).

Comparison of sample force-extension profiles and mechanical strengths of protein L I60V and I60F (Fig. 5a and c) shows that the mechanical stability of protein L changes markedly from I60V to I60F: substitution of valine to phenyalanine at this position results in an increase in mechanical strength of 108 pN. However, this value represents a lower estimate for the mechanical enhancement gained from this point mutation. Figure 5a shows that the relative stability of protein L to I27 changes from I60V to I60F. In ((I27-pL^I60V^)_3_I27), each protein L domain is most likely to unfold before any of the I27 domains have unfolded, whilst for ((I27-pL^I60F^)_3_I27), each protein L domain is most likely to unfold after all of the I27 domains have unfolded. As discussed above, changes in the order of unfolding of domain types can significantly affect the apparent strength of each protein within the heteropolymer. Consequently, the mechanical properties of homopentameric analogues of these variants were calculated by Monte Carlo simulations using values of *k*_u_ and *x*_u_ previously determined by fitting the speed dependence of the unfolding force for heteropolymeric constructs as described above (Fig. 3). These data (Table 1) reveal that a single point mutation in the hydrophobic core of protein L changes the mechanical strength by ∼ 117 pN.

## Discussion

We have used protein engineering techniques coupled with single-molecule force spectroscopy to visualise the mechanically induced unfolding transition state for the small, topologically simple protein L. Of five variants studied, only I60V showed significant differences to WT protein L. Consequently, the mechanical transition state for protein L is highly native-like. To date, similar analyses have been reported for only two Ig-like β-sandwich proteins I27 (I-set Ig I27 from titin)^10^ and the third fibronectin type 3 domain from tenascin (TNfn3).^34^ Despite their similar folds, these proteins were found to have distinct mechanical transition states. Similar to protein L, high Φ values were found for most positions probed in I27. However, for TNfn3, a broader range of Φ values were found because the mechanical unfolding transition state for TNfn3 occurs after the formation of an aligned intermediate state involving core rearrangement.

The large decrease in unfolding force for I60V is surprising given that similar hydrophobic deletions elsewhere in the protein have little effect. Previous simulations of the mechanical extension of protein L showed that unfolding occurs by the shearing and abstraction of one structural sub-domain from the rest of the structure (red and green regions, respectively, Fig. 1a). The mechanical strength of ‘brittle’ proteins such as protein L, ubiquitin and I27 may thus be modulated by the number of hydrophobic contacts made across the sub-domains that are sheared apart. The large decrease in unfolding force and change in the placement of the unfolding transition state for I60V accords with this hypothesis. For example, L10 and I60 are located on strands I and IV of protein L, respectively (Fig. 1a). L10 makes contacts with nine other side chains in the hydrophobic core, seven of which reside within sub-domain 1, consistent with the finding that truncating this side chain has little effect on mechanical stability. For I60, of 10 contacting side chains in the core, 6 occur between the sub-domains, rationalising the dramatic effect of truncation of this side chain on the mechanical phenotype. These effects are not simply related to the degree of thermodynamic destabilisation (Table 1) but appear instead to be critically dependent on the precise interactions that are disrupted. I60 thus acts as a ‘linchpin’ that locks the native state into a mechanically strong structure. Removal of this key mechanical residue transfers the role of linchpin interactions to other side chains, resulting in a softening of the protein and an increase in *x*_u_. A similar mutational analysis^10^ undertaken on I27 found that reduction in the hydrophobic side chain of core residues (I23A, L58A and F73L) had no effect on the mechanical resistance of this domain because the mechanical clamp occurs between strands A′ and G. Similar mutations in these strands (V13A and V86A) were found to mechanically destabilise the protein significantly. By contrast, the mechanical transition state for TNfn3^34^ occurs after the formation of an intermediate. For this protein, mechanical destabilisation was observed for variants in the core (I8A and V70A) and throughout the structure (Y68F and T90A). Thus, core packing can play a vital role in determining the mechanical response of a protein but only if these contacts occur in force-bearing regions of the protein. To test this hypothesis, we created hydrophobic enhancement variants that were thermodynamically favourable by filling a small cavity within the core of our WT protein by either crossing the putative mechanical interface between sub-domains (I60F), remaining within one sub-domain (L10F), or simply reverting to the hydrophobic packing present in true WT protein L (W47Y protein L). Analysis of these variants clearly demonstrated that, similar to the hydrophobic reduction variants, the location of interactions was key to determining their mechanical properties.

In contrast to previous data, we have shown that a conservative mutation of a single site can fundamentally alter the mechanical strength of a protein: substitution of valine for phenyalanine at position 60 results in an increase in the mechanical strength of at least 2-fold (108 and 216 pN at 447 nm s^− 1^). The magnitude of the mechanical strengthening of protein L, which results from a single mutation, is remarkable when compared with other reports of increases in the mechanical resistance of proteins by mutagenesis of core residues. For example, substitution of seven residues in the core of GB1 with their equivalents from a hyperthermophilic variant resulted in a 17% (30 pN) increase in mechanical strength.^37^ For I27 and the 10th domain of human fibronectin (FNfn10), substitution of 12 and 15 core residues with those found at analogous positions of mechanically stronger homologues (I32 and TNfn3) enhanced their mechanical strength by ∼ 47% and 20% (or ∼ 81 and 21 pN) at 400 and 1000 nm s^− 1^, respectively.^11,38^ Whilst not using conservative mutations, the Li group have reported two elegant methods to enhance protein mechanical strength. Under mechanical extension, Top7 unfolds by the shearing apart of the weaker of two mechanical interfaces. Blocking this pathway by introduction of a disulfide bridge led to an increase of ∼ 30 pN.^39^ In a second method, insertion of bi-histidine metal chelation sites at different sites in protein GB1 led to an increase of ∼ 120 pN in the presence of 4 mM Ni^2+^. However, in this case, the apo forms of GB1 were destabilised by up to 65 pN.^40^

Modulating the hydrophobic contacts across mechanical interfaces may be a generic method by which the mechanical strength of proteins can be tuned and this method may be especially appropriate for those proteins with highly native-like mechanical transition states. Whilst our design strategy was facilitated by the presence of a hydrophobic cavity within Y47W protein L, the same effect can be achieved by complementing increases and decreases in hydrophobic side-chain volume at suitable sites within the target protein.

It has been shown that the topology of a protein can be used to rank the mechanical response of proteins with different folds.^3,4^ This coarse-grained approach cannot explain the large variation seen within highly homologous proteins expected to have almost identical structures. The finding that the precise packing of a particular core residue plays a key role in determining protein mechanical strength provides a mechanism by which nature can evolve a varied mechanical response without a gross change of topology. Force resistance may thus play an important role in the evolution of the amino acid sequence of force-bearing or responsive proteins. Modulating the hydrophobic contacts across mechanical interfaces by complementary changes in hydrophobic side-chain volume of core residues may be a generic but facile method by which the mechanical strength of proteins can be tuned.

## Materials and Methods

### Heteropolymeric protein construction, over-expression and purification

The sequence of protein L used in this study and in previous studies^7^ is a Y47W variant constructed to facilitate protein folding studies.^19^ Heteropolymeric proteins were constructed using a cassette strategy as described previously.^29^ Briefly, cassettes suitable for insertion into positions 2, 4 and 6 of a previously constructed concatenated gene encoding heptameric I27 were generated and point mutations were introduced using QuikChange site-directed mutagenesis (Stratagene). After verification by DNA sequencing, I27 cassettes at positions 2, 4 and 6 were sequentially substituted with their protein L analogues. The final construct has four (C47S, C63S) I27 domains alternating with three protein L domains with each domain separated by an unstructured linker:

MHHHHHHSS(I27)VEARGG(proteinL)GLIEAR(I27)LSSARGG(proteinL)GLIERARGG(I27)LIRGRGG(proteinL)GLIVQAR(I27)CC

For over-expression by auto-induction, plasmids encoding heteropolymeric constructs were transformed into *Escherichia coli* BLR(DE3) (Novagen) and cultured as described previously.^41^ All proteins were purified using sequential nitrilotriacetic acid affinity and size-exclusion chromatographies as described previously.^7^

### Mechanical unfolding and data extraction

Mechanical unfolding experiments were performed using an MFP-SA or MFP-3D-SA (Asylum Research Inc., Santa Barbara, CA, USA) using standard protocols.^7,29^ An aliquot (0.05 mg) of the appropriate freeze-dried protein was dissolved into 0.25 ml phosphate-buffered saline, pH 7.4, and centrifuged in a microfuge at 13,000 rpm for 5 min, and the supernatant was retained. The protein solution was further diluted with phosphate-buffered saline as appropriate, and 100 μl was applied onto a freshly template-stripped gold surface. After thermal equilibration, force-extension profiles were accumulated at 100, 270, 700 and 2000 nm s^− 1^. Data were filtered, binned and analysed as described previously.^7,21,29^ A summary of the unfolding data for all variants is included in Supplementary Information.

### Monte Carlo simulation

Monte Carlo simulations were carried out using a two-state model described previously^7^ with a slight modification for heteromeric polyproteins. In the experiment, it is assumed that the C-terminus of the polyprotein was linked to the gold surface by S–Au bond and the cantilever randomly picked up the other end by using physical adsorption. In the simulations, the number of domains picked up by the cantilever was randomly chosen to be within the range of 2 (including only one pL) to 7 (three protein L domains and four I27 domains) at the start of each pulling simulation. Then, the cantilever was retracted at a constant rate and the applied force (*F*) was calculated using the WLC model at each extension. The unfolding probabilities of pL and I27 within time step (d*t*) were *N*_f_^pL^*k*_u_^pL^exp(*Fx*_u_^pL^/*k*_B_*T*)d*t* and *N*_f_^I27^*k*_u_^I27^exp(*Fx*_u_^I27^/*k*_B_*T*)d*t*, respectively, where *N*_f_ is the number of folded domains of each domain type, *k*_u_ is the unfolding rate constant at zero force of each domain type and *x*_u_ is the distance between the folded state and the transition state in the mechanical unfolding coordinate of each domain type. If unfolding of either domain occurred, the contour length was increased by d*L* (18.55 nm for pL and 28 nm for I27). The calculation was continued using the new contour length until all domains unfolded. This process was repeated 1000 times, and the modal force was calculated from the unfolding force histogram.

A matrix of *k*_u_^pL^ and *x*_u_^pL^ values were sequentially tested pairwise using fixed values for *k*_u_^I27^ and *x*_u_^I27^ of 0.0028 s^− 1^ and 0.26 nm, respectively, to calculate *k*_u_ and *x*_u_ for WT protein L and its variants. For each pair of *k*_u_^pL^ and *x*_u_^pL^, the mode force at each pulling speeds (100, 270, 700 and 2000 nm s^− 1^) was calculated and compared to the experiment. The values of *k*_u_ and *x*_u_ of pL mutants were determined as the pair that gives the minimum of the sum of squares of the differences in mode force between the simulation and the experiment.

### Φ-value analysis

ΔΔ*G*°_UN_ values were the difference of the average of two values for Δ*G*_UN_ of WT protein L and its variants described previously.^42^

Two different methods were used to calculate ΔΔ*G*°_TS–N_ from mechanical unfolding data.^33^ In the first method, ΔΔ*G*°_TS–N_ was calculated directly from the speed dependence of unfolding force for each protein:

$$\Delta \Delta G_{\text{TS}-N}^{\text{WT}-\text{MUT}}=RT\left(f_{\text{WT}}-f_{\text{MUT}}\right)/m$$

where *m* is the average gradient of the weighted best-fit lines to the speed dependence of the unfolding force for WT, L10A, F22A, A37G and V51A and *f*_WT_ and *f*_MUT_ are the unfolding forces of WT and mutant protein L, respectively, at 447 nm s^− 1^. Errors were propagated using an error of 5% in the force values based on the variation in average unfolding force values of I27 in L10A, F22A, A37G and V51A. Errors on values for ΔΔ*G*_UN_ are derived from the difference in estimated values of Δ*G*_UN_ for WT and each variant using the different methods described previously.^42^ In the second method, values of *k*_u_^0F^ obtained from the MC simulation were used to calculate ΔΔ*G*_TS–N_ as:

$$\Delta \Delta G_{\text{TS}-N}^{\text{WT}-\text{MUT}}=-RT\ln\left(k_{u}^{0\text{F,WT}}/k_{\text{u}}^{0F,\text{MUT}}\right)$$

Φ_F_ can then be calculated:

$$\Phi_{F}=1+\frac{RT\ln\left(k_{u}^{0\text{F,WT}}/k_{u}^{0\text{F,MUT}}\right)}{\Delta \Delta G_{\text{UN}}}$$

The results for each method are shown in Table 1.

### Equilibrium denaturation

Monomers of protein L I60V and I60F were constructed by QuikChange site-directed mutagenesis (Stratagene) using a previously described vector encoding a (His)_6_-tagged (Y47W) protein L monomer.^19,43^ After verification of the DNA sequence, each protein was over-expressed as described previously.^21^ Monomers were purified to homogeneity using histidine Ni-NTA affinity chromatography^21^ followed by cation exchange (SP-HP 5 ml, GE Healthcare). After elution, the protein was dialysed into ultrapure water (Ondeo Industrial Solutions) and freeze-dried. Fluorescence spectroscopy was performed on a Photon Technology International QM-1 spectrofluorimeter (PTI, UK) at 20 °C using a 1-ml quartz Hellma cuvette. Monomers of L10F and I60F protein L were prepared from stocks containing 10 μM of protein in 50 mM sodium phosphate at pH 7 and 2 mM ethylenediaminetetraacetic acid with increasing levels of guanidinium hydrochloride (GdmHCl). The stock concentration of GdmHCl was calculated by measurement of the refractive index of the stock solution. After equilibration for at least 4 h, fluorescence was excited at a wavelength of 280 nm and monitored at the point of maximum difference between the native and denatured states (typically 321–325 nm, depending on the mutant observed). The fluorescence emission intensity was measured for 60 s. After signal averaging, the intensity was plotted as a function of GdmHCl concentration and the data were fitted to a two-state transition as described previously.^44^ To compare the denaturation profiles of the variants with those obtained previously for WT protein L,^19^ the raw data were converted to fraction population of native molecules.^45^

## Figures and Tables

**Table 1:** Thermodynamic, kinetic and mechanical stability of WT protein L and its variants

Variant	Position	ΔΔ*G*°_F–U_ (kJ mol^− 1^)^a^	*k*_u_ (s^− 1^)^b^	Φ_F_^D^^c^	Δ*F*_447_ (pN)^d^	Δ*F*_447_^MC^ (pN)^e^	*k*_u_^0F^ (s^− 1^)^f^	*x*_u_ (nm)^f^	Φ_F_^F^^g^	Φ_F_^F^^h^
WT	—	—	0.1	—	—	—	0.02	0.26	—	—
L10A	Strand I	− 12.3 ± 0.8	1.2	0.43	− 5 ± 10	− 9	0.04	0.25	0.95 ± 0.04	0.9
F22A	Strand II	− 20.8 ± 3.1	14.5	0.41	0 ± 10	− 1	0.05	0.23	1.00 ± 0.03	0.9
A37G	Helix	− 13.7 ± 0.6	13.7	0.11	0 ± 10	− 6	0.08	0.22	0.99 ± 0.04	0.8
V51A	Strand III	− 4.6 ± 0.1	0.3	0.19	− 8 ± 10	− 13	0.03	0.27	0.78 ± 0.11	0.8
I60V	Strand IV	− 0.7 ± 0.1	0.55	0.22	− 36 ± 9	− 36	0.002	0.46	0.33 ± 0.08	ND
L10F	Strand I	2.6 ± 0.5	ND	ND	13 ± 11	18	0.03	0.22	ND	ND
I60F	Strand IV	5.5 ± 0	ND	ND	72 ± 13	81	0.01	0.17	ND	ND

ND, not determined.

a ΔΔ*G*°_F–U_ (Δ*G*°_F–U_^MUT^ − Δ*G*°_F–U_^WT^) is the mean of two values (ΔΔ*G*_F–U_^Cm^, ΔΔ*G*_F–U_^2M^) as described previously.^20^ Experimental data for L10F and I60F are shown in Fig. 4.

b Intrinsic unfolding rate constant measured by chemical denaturants taken from Ref. 20. Note that these values are determined at 2 M GdmHCl (*k*_u_^2M^), pH 7, and 295 K.

c Folding Φ values measured by chemical denaturants taken from Ref. 20.

d The difference in unfolding force between each protein L variant and WT at an extension speed of 447 nm s^− 1^. Δ*F*_447_ = *F*_MUT_^447^ − *F*_WT_^447^. Note that *F*_WT_^447^ = 144 pN.

e The difference in unfolding force (Δ*F* = *F*_MUT_–*F*_WT_) between pentameric constructs of protein L variants and WT at an extension speed of 447 nm s^− 1^ calculated by Monte Carlo simulations (Materials and Methods). *F*_WT_^447^ = 134 pN.

f Intrinsic unfolding rate measured by force (*k*_u_^0F^) and *x*_u_ calculated from Monte Carlo simulations (Materials and Methods).

g Φ_F_^F^ calculated using unfolding force for each variant at 447 nm s^− 1^ (Materials and Methods).

h Φ_F_^F^ calculated using *k*_u_^0F^ values derived from Monte Carlo fits (Materials and Methods).

## Appendix A

Supplementary materials Summary of unfolding data for ((I27-pL^WT^)_3_I27). Summary of unfolding data for ((I27-pL^L10A^)_3_I27). Summary of unfolding data for ((I27-pL^F22A^)_3_I27). Summary of unfolding data for ((I27-pL^A37G^)_3_I27). Summary of unfolding data for ((I27-pL^V51A^)_3_I27). Summary of unfolding data for ((I27-pL^I60V^)_3_I27). Summary of unfolding data for ((I27-pL^L10F^)_3_I27). Summary of unfolding data for ((I27-pL^I60F^)_3_I27).
