# Supplementary material for: Identification of a Mechanical Rheostat in the Hydrophobic Core of Protein L
Source: J Mol Biol. 2009 Oct 16;393(1):237–48. doi: 10.1016/j.jmb.2009.08.015 (PMC2796179; doi:10.1016/j.jmb.2009.08.015)
Supplement: Supplementary materials — Summary of unfolding data for ((I27-pLWT)3I27). Summary of unfolding data for ((I27-pLL10A)3I27). Summary of unfolding data for ((I27-pLF22A)3I27). Summary of unfolding data for ((I27-pLA37G)3I27). Summary of unfolding data for ((I27-pLV51A)3I27). Summary of unfolding data for ((I27-pLI60V)3I27). Summary of unfolding data for ((I27-pLL10F)3I27). Summary of unfolding data for ((I27-pLI60F)3I27). [file mmc1.doc]

**Supplementary Information**

Table A1: summary of unfolding data for (I27-pL^WT^)_3_I27).

Table A2: summary of unfolding data for (I27-pL^L10A^)_3_I27).

Table A3: summary of unfolding data for (I27-pL^F22A^)_3_I27).

Table A4: summary of unfolding data for (I27-pL^A37G^)_3_I27).

Table A5: summary of unfolding data for (I27-pL^V51A^)_3_I27).

Table A6: summary of unfolding data for (I27-pL^I60V^)_3_I27).

Table A7: summary of unfolding data for (I27-pL^L10F^)_3_I27).

Table A8: summary of unfolding data for (I27-pL^I60F^)_3_I27).
